# Challenges and Opportunities for Population Health Partnerships

**Published:** 2010-10-15

**Authors:** Stephen M. Shortell

**Affiliations:** School of Public Health, University of California, Berkeley

The Mobilizing Action Toward Community Health (MATCH) articles in this issue of Preventing Chronic Disease discuss ideas, policies, and practices that can be used to produce a healthier population in the United States and globally. The articles pose the following questions: 1) How do we best measure long-term wellness at the population level?, 2) How do we provide incentives to organizations to accomplish better population health?, and 3) How can effective cross-sector partnerships be formed and implemented to help accomplish the task?

The articles in this issue have done a good job, for the most part, of summarizing what we know or at least what we think we know about successful partnerships. They highlight the many challenges of forming cross-sector partnerships, given the different goals, objectives, and cultures of potential partners. They also provide ideas and evidence for overcoming some of these challenges; the importance of leadership, governance, measurement and accountability, focus, and trust are all emphasized. What these discussions lack is consideration of the interrelated practices and behaviors that may prove useful, given widely varying community contexts — geographic, political, economic, and social. Some examples of what is missing that I suggest as a basis for further discussion include the following:

1. Partnerships need to be both internally and externally aligned. Partners should achieve domain consensus among themselves with sufficient overlap of goals and should understand what is expected of the partnership by external groups.
2. The partnership should gain legitimacy and credibility within the community. Drawing on the developing literature on social capital would improve this process (1).
3. Partnerships can gain legitimacy by understanding their centrality in the political economy of the community. Social network concepts involving direct and indirect ties, the strength of ties, network density, and structural holes are relevant.
4. Every partner has a core competence and comparative advantage. Partnerships can fail because individual members either overestimate or underestimate their comparative advantage and misdiagnose their core competence.
5. Leadership should be explored more fully: the kind of leadership needed, the kind of partnership that can deliver it, and the stage of the partnership’s life cycle that is best suited for it. The role of individual leadership versus organizational leadership should be discussed (2).
6. Forming a partnership has a transaction cost. The literature on transaction cost economics originally developed by Williamson may be relevant (3).
7. The process of selecting partners, including tradeoffs and timing, should be more fully explored.
8. Population health improvement can be perceived as simply a resource for organizations to advance their own agenda and cause.

In addition to pursuing these ideas, we may take the following actions to improve population health. First, we may consider the Healthy People 2020 objectives, which will depart from the past by emphasizing the underlying environmental and social determinants of health. They may provide a stimulus and framework for considering population health improvement.

Second, we should consider population health improvement in the context of health care delivery system reform. The article by Hester, for example, highlights the developing Vermont experience with accountable care organizations (ACOs) (4). These entities are accountable for the cost and quality of care provided to a given population of patients; they can be linked to population health improvement objectives by expanding the chronic care model to recognize community contributions to health. A promising approach is to recognize the patient-centered medical home (PCMH) model of primary care delivery as the foundation for ACOs (5). Payment reforms could achieve positive health outcomes by using the framework of ACOs and PCMHs. For example, one approach would be to provide bundled or capitated payments to public health departments that would in turn work with ACOs and PCMHs to provide cost-effective care to defined populations.

Third is the concept of community health management systems (CHMS) that would be organized along the lines of local security and exchange commissions as quasi-administrative, publicly accountable bodies (6). The CHMS may be a partnership or coalition of the local health department; community organizations; ACOs made up of local hospitals, physician practices, and other provider entities; and related health care providers. CHMS would have 3 functions: 1) assess and prioritize the health needs of the population from a multisectoral approach; 2) organize the community’s assets, resources, and competencies to deliver the needed services; and 3) be held clinically and fiscally accountable for the health outcomes produced. They would deliver an annual report to relevant political bodies in the community. The success of the CHMS and related concepts depends on the availability of relevant population-based metrics for health outcomes and on payment incentives that encourage integration of the multiple sectors involved in producing population health.

Incorporating these suggestions could advance our understanding of effective cross-sector population health partnerships. Expansion of the knowledge base will help to promote the spread of such partnerships across the country. National health care reform legislation provides additional impetus and opportunities for such achievement because it emphasizes ACOs and PCMHs by providing financial incentives for their development and increases funding for health promotion and wellness programs. 
